# Complex multisource sound induces greater neurodegeneration in neonatal rat brain than single-source sound

**DOI:** 10.3389/fnsys.2026.1767882

**Published:** 2026-01-28

**Authors:** İskender Samet Daltaban, Mehmet Dumlu Aydın, Eylem Eren Eyüpoğlu, Elif Demirci, Aybike Aydin Okuyan, Mehmet Emin Demir

**Affiliations:** 1 Department of Neurosurgery, Alife Hospital, Ankara, Türkiye; 2 Department of Neurosurgery, Faculty of Medicine, Atatürk University, Erzurum, Türkiye; 3 Department of Neurosurgery, Ministry of Health, Ankara Oncology Education and Research Hospital, Ankara, Türkiye; 4 Department of Pathology, Faculty of Medicine, Atatürk University, Erzurum, Türkiye; 5 Faculty of Medicine, Istanbul University Cerrahpasa, Istanbul, Türkiye; 6 Department of Internal Medicine, School of Medicine, Atılım University, Ankara, Türkiye

**Keywords:** amygdala, hippocampus, loudspeakers, neurotoxicity, newborn brain, noise-induced neurodegeneration, sound interference, stereology

## Abstract

**Background:**

Excessive noise exposure is a known environmental health hazard linked to neurological injury and cognitive deficits. Whether complex sound waveforms from multiple sources exacerbate brain damage compared to a single-source noise of equal intensity remains unclear. We investigated the effects of identical music played either through one or four loudspeakers on the developing brain of newborn rats.

**Methods:**

Forty-one newborn Sprague–Dawley rat pups (both sexes), along with their dams, were randomly assigned to three groups: control (no noise, *n* = 6), single-speaker exposure (*n* = 15), and multi-speaker exposure (*n* = 20). From postnatal day 0 to 30, the exposure groups were subjected to an 8-min music track (~85 dB SPL) either via one loudspeaker (simple waveform) or simultaneously via four loudspeakers (complex interfering waveform), six times daily at 4-h intervals. Sound intensity was calibrated at the cages with a sound-level meter. All procedures followed ARRIVE guidelines and the EU Directive 2010/63/EU for animal research, with institutional ethical approval. After 1 month, rat brains were examined histologically. Unbiased stereology was used to estimate neuronal densities in the temporal lobe (including amygdala and hippocampal dentate gyrus). Immunohistochemistry for neuron-specific enolase (NSE), glial fibrillary acidic protein (GFAP) and TUNEL assay (terminal deoxynucleotidyl transferase dUTP nick-end labeling) was performed to identify neuronal integrity, astroglial response, and apoptosis, respectively. Outcome measures were degenerated (TUNEL-positive) neuron densities and histopathological lesions. Statistical comparisons were made using Student’s *t*-tests or ANOVA and chi-square tests (with *p* < 0.05 considered significant).

**Results:**

Eight of 20 pups (40%) in the multi-speaker group died during the exposure period, compared to 5/15 (33%) in the single-speaker group and 3/6 (50%) in controls (differences not statistically significant). Maternal rats exhibited agitation, stress behaviors, and weight loss under noise; some eventually ceased escape attempts (habituation/helplessness behavior) in both noise-exposed groups. Histologically, the multi-speaker exposure caused more severe brain injury than the single-speaker exposure. Pups in the multi-speaker group showed frequent subarachnoid hemorrhages and cortical microvascular bleeding in the temporal lobes, whereas these lesions were mild or infrequent in the single-speaker group and absent in controls. Neurons in noise-exposed brains displayed morphological signs of degeneration (shrunken, angulated cell bodies with pyknotic nuclei and condensed cytoplasm), which were markedly pronounced in the multi-speaker group. Stereological cell counting revealed a significant increase in apoptotic neuron density in both sound-exposed groups, with the multi-speaker group highest. For example, in the hippocampal dentate gyrus, the mean density of TUNEL-positive (degenerating) neurons was 13,450 ± 1,560 per mm^3^ in the multi-speaker group vs. 7,600 ± 980 per mm^3^ in the single-speaker group and only 200 ± 34 per mm^3^ in unexposed controls (*p* < 0.05). In the amygdala, apoptotic neuron density averaged 3,460 ± 863 per mm^3^ (multi-speaker) vs. 1,470 ± 285 (single-speaker) and 1,321 ± 234 (control), with the multi-speaker group showing a significantly higher burden of neuronal cell death (*p* < 0.005 for complex vs. simple waveforms). Correspondingly, multi-speaker exposed brains showed intense immunostaining for NSE and GFAP fragmentation, indicating widespread neuronal loss and reactive astroglial injury, whereas single-speaker exposure caused only moderate changes.

**Conclusion:**

Identical musical noise caused substantially more neurodegeneration in the developing brain when delivered as complex wave interference from multiple speakers rather than as a single-source sound of the same intensity. Complex multisource waveforms appear to amplify the harmful effects of noise on neonatal brain tissue, likely through interference-driven pressure fluctuations. These findings have clinical and public health implications, suggesting that current noise exposure guidelines should consider not only sound intensity and duration but also the acoustic complexity and source configuration, especially to protect vulnerable populations such as infants and children from high-intensity multisource noise environments.

## Introduction

Environmental noise is increasingly recognized as a neurotoxic stressor affecting brain health and development (Li et al., 2022; Liu et al., 2025). Chronic exposure to loud noise can provoke a range of adverse neurological outcomes, including cognitive impairment, neuroinflammation, and even acceleration of neurodegenerative processes (Li et al., 2022; Liu et al., 2025; Lee et al., 2025). Epidemiological studies have linked sustained community noise to poorer mental health and cognition, and laboratory research indicates that intense noise trauma may damage the brain in ways analogous to traumatic brain injury (TBI) (Li et al., 2022; Liu et al., 2025; Säljö et al., 2003). For example, a single impulse noise at extreme intensity (~200 dB) can transiently increase neuronal membrane permeability and trigger diffuse brain injury hallmarks (cytoskeletal disruption, apoptosis, glial activation) without overt hemorrhage (Säljö et al., 2003). Repeated noise above ~85 dB has been shown to induce lasting synaptic and neuronal losses in animal models. Noise exposure during critical developmental periods is of special concern: prenatal noise has been found to impair hippocampal neurogenesis and learning in offspring, and brief intense noise in early postnatal life can lead to permanent structural changes in the auditory system (Kim et al., 2006; Ouda et al., 2016). Yet despite such evidence, many people, including children and pregnant women, are routinely exposed to high levels of noise in daily life, such as in urban environments or entertainment venues (European Environment Agency, 2020).

Modern urban and recreational settings often feature complex acoustic environments with multiple loudspeakers or sound sources (Basner et al., 2014). For instance, nightclubs, concerts, or large public events can reach sound levels well above 100 dB with multiple speakers operating simultaneously in enclosed spaces (Williams et al., 2010). In these scenarios, the same sound (e.g., music) is emitted from several sources placed at different locations. Physically, multiple sound waves interacting in space produce interference patterns: constructive and destructive interference leads to a complex waveform with fluctuations in amplitude and frequency content that differ from the original simple waveform (Kenneth and Janssen, 2019; Oxenham, 2018; Zhang and Piao, 2021). By Fourier analysis, a musical signal from one source can be represented as a relatively stable combination of frequencies, whereas that same signal emitted from multiple sources will superimpose to create new composite waveforms with spatially heterogeneous intensity peaks and troughs (Chai et al., 2021). We hypothesized that such complex interfering waveforms might impose greater stress on neural tissue than a single-source sound of equivalent average intensity, perhaps by generating higher instantaneous pressure gradients or a broader spectrum of frequencies impacting the brain (Säljö et al., 2003; Chai et al., 2021; Manohar et al., 2022). However, this hypothesis had not been directly tested (Oxenham, 2018; Chen et al., 2016; Jáuregui-Huerta et al., 2011). Noise regulations and safety guidelines generally focus on overall sound pressure level and exposure duration, without accounting for whether the noise originates from one coherent source or many asynchronous sources (Basner and McGuire, 2018; Kempen et al., 2018).

This study aimed to elucidate the potential role of acoustic waveform complexity as an independent determinant of neurobiological vulnerability in the developing brain. Specifically, it sought to determine whether multi-source, interference-based acoustic fields impose greater neural stress than single-source sounds of equivalent intensity. The ultimate objective was to provide mechanistic insights that could inform evidence-based refinement of existing noise-exposure standards by incorporating waveform complexity as a critical risk parameter alongside conventional decibel and duration metrics.

## Materials and methods

### Animals and ethical compliance

This study used neonatal Sprague–Dawley rats and was conducted in strict accordance with international guidelines for animal research. All experimental protocols were approved by the Institutional Animal Care and Use Committee (Atatürk University Faculty of Medicine Ethical Committee, Approval No. B.30.2. ATA.0.23.85–21, dated 25/03/2011). The study design and reporting adhere to the ARRIVE 2.0 guidelines for animal experiments and comply with the U.S. National Institutes of Health Guide for the Care and Use of Laboratory Animals (2011) as well as the European Directive 2010/63/EU on the protection of animals used for scientific purposes (Percie du Sert et al., 2020). All efforts were made to minimize animal suffering and to use the minimum number of animals necessary to achieve statistical significance.

A total of 41 newborn rat pups (from several litters) were used, along with their nursing dams. Newborns of either sex were included (sex was not determined at birth and outcomes were not analyzed by sex), and pups were randomly distributed across groups. Sample size was determined based on ethical considerations and ARRIVE guidelines, prioritizing the minimum number of animals required to demonstrate histopathological differences, rather than a formal *a priori* power analysis. All efforts were made to minimize animal use and suffering in accordance with EU Directive 2010/63/EU. No pre-exposure auditory screening (e.g., ABR or DPOAE) was performed, as pups were studied from birth and handling was minimized. However, pups originated from healthy breeding colonies with no known auditory deficits, and random distribution across multiple litters was used to reduce litter-specific bias. The pups were housed with their mothers in standard polycarbonate cages in a temperature-controlled room (22 ± 1 °C) on a 12:12 h light/dark cycle. Mothers had free access to food and water. To ensure maternal care and feeding were not disrupted, dams remained with their litters throughout the experiment, and each dam with her pups constituted a single experimental unit in a separate exposure chamber.

### Experimental groups and noise exposure paradigm

The pups were randomly divided into three experimental groups:

- Control group: Six pups (from two litters) were reared in a quiet environment with no deliberate noise exposure, apart from normal ambient room sounds (which were kept under 50 dB). This group controlled for handling and environmental conditions without the specific music stimulus.
- Single-speaker group (simple waveform): 15 pups (from four litters) were exposed to a musical stimulus played through a single loudspeaker, producing a relatively simple acoustic waveform.
- Multi-speaker group (complex waveform): 20 pups (from five litters) were exposed to the same musical stimulus played simultaneously through four identical loudspeakers placed around the environment, producing an interferent complex waveform.

**Figure 1:**
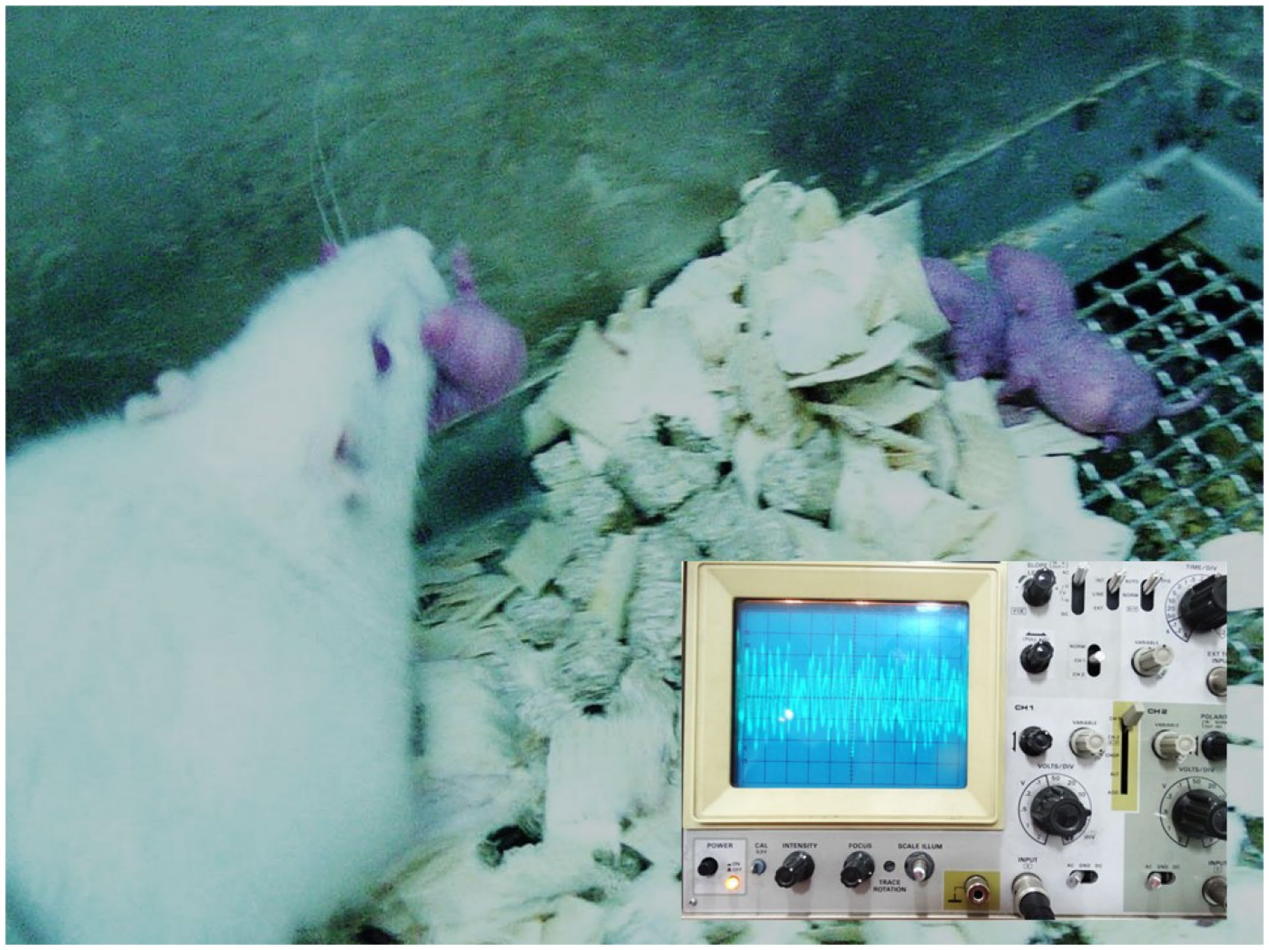
A sound analyzer used to physically characterize the sound waves of the musical stimulus delivered to neonatal rats is shown. A mother rat is seen attempting to hide her pups in response to the noise exposure. The impulse wave–producing apparatus used for waveform analysis is visible in the lower right corner of the image.

For the noise exposure, the auditory stimulus was a commercially available dance/disco music track containing both vocal and instrumental elements (mixed vocals/instruments), 8 min in duration, with a broad frequency range of approximately 300 Hz–8 kHz (Figure 1). This frequency range was intentionally chosen to model common human music exposure environments rather than to encompass the full auditory range of rats, which extends into ultrasonic frequencies. Importantly, although ultrasonic components were not included, the selected bandwidth falls within the most sensitive hearing range of rats and was sufficient to induce marked neurobiological effects. The same music track was used for both the single- and multi-speaker groups. Sound delivery was achieved using a high-fidelity audio system consisting of a CD player and an amplifier (Pioneer PD-310 CD player and Yamaha P4500 amplifier). In the single-speaker condition, one standard full-range loudspeaker was mounted centrally above the cage (~50 cm from the pups) to ensure uniform exposure. In the multi-speaker condition, four identical loudspeakers were positioned equidistantly around the cage so that the music was played simultaneously from all four sources. This configuration was designed to ensure that the combined sound pressure level at the center of the cage matched that of the single-speaker condition, thereby equalizing average intensity while allowing spatial and temporal interference patterns to occur.

**Figure 2:**
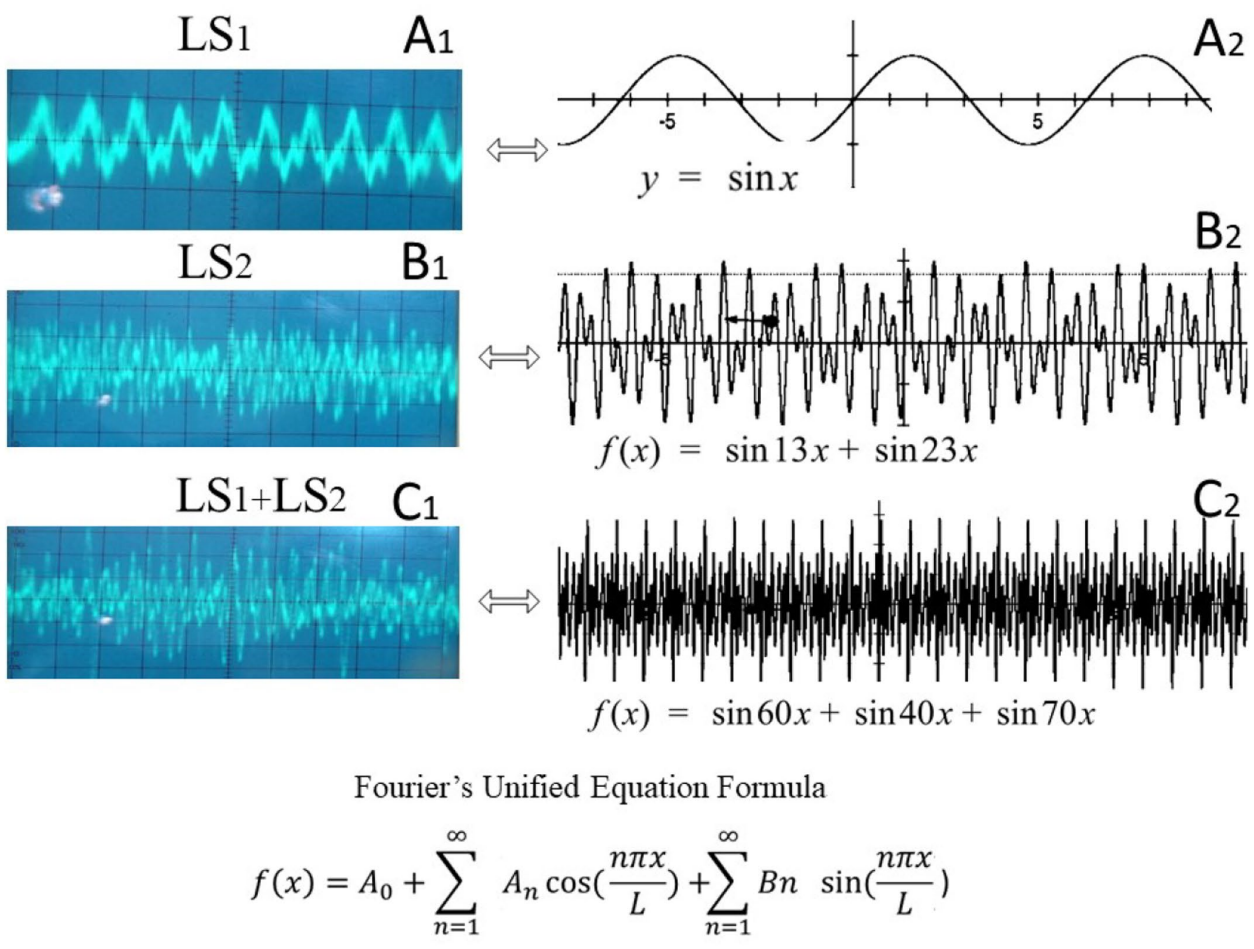
Acoustic interference patterns generated by single- and multi-speaker sound delivery. Sound waves emitted from a single loudspeaker (LS1) shown in **A1** and the corresponding sine-wave equation is shown in **A2**. Sound waves emitted from a second single loudspeaker (LS2) is shown in **B1** and the corresponding sine-wave equation is shown in **B2**. Sound waves emitted simultaneously from both speakers (LS1 + LS2), demonstrating constructive and destructive interference is shown in **C1**, and the corresponding combined sine-wave equation is shown in **C2**. The formal combined Fourier wave equation illustrates how instantaneous pressure fluctuations and the physical properties of musical sound arise when identical waveforms interact in space.

Sound calibration: We used a precision sound level meter (AZ Instrument Corp., model 8,926) with A-weighting to calibrate the noise intensity in each setup. The target sound pressure level (SPL) was 85 dB (A-weighted) at the height of the cage floor (approximately ear level of the pups). For the single-speaker group, the amplifier volume was adjusted to produce ~85 dB SPL consistently throughout the cage. For the four-speaker group, each speaker was set at a proportionally lower output such that their combined sound field at the cage center was ~85 dB SPL (±5 dB). During simultaneous multi-speaker playback, naturally, localized spots of constructive interference could reach higher instantaneous peaks (we measured brief spikes up to ~90–95 dB in some positions), while destructive interference created troughs below 85 dB elsewhere. The waveform characteristics were monitored with an oscilloscope (Leybold-Heraeus 40 MHz digital oscilloscope) and a sensitive microphone (50 Hz–15 kHz range) to verify the presence of interference patterns. The principal frequency content of the music was around 2.5 kHz (fundamental frequency determined by measuring a ~ 0.4 ms period on the oscilloscope, corresponding to 2,500 Hz). We confirmed that the *single-speaker* playback produced a relatively consistent sinusoidal waveform envelope for the music, whereas the *multi-speaker* playback yielded a more complex, fluctuating waveform (demonstrated by overlapping wave traces and a Fourier sum of the individual speaker outputs) (Figure 2). All exposures were conducted in acoustic isolation chambers to prevent external noise contamination and to ensure that control animals were not exposed to the music.

Exposure schedule: Rats in the noise groups underwent this exposure six times a day, at 4-h intervals (e.g., 6 sessions spaced evenly over 24 h). Each session consisted of playing the 8-min music track once. Thus, total exposure per day was 48 min of music. This daily regimen was repeated for 30 consecutive days, spanning roughly the first postnatal month (a period equivalent to human early infancy in terms of brain development). The control group rats were housed under identical conditions (including being placed in similar cages and chambers) but with no music played; handling and timing were kept consistent (e.g., control cages were taken in and out of chambers on the same schedule without sound). Pups remained with their mothers throughout to ensure normal feeding and social interaction. The mothers in the noise groups were unrestrained and free to care for pups during exposure; they thus also experienced the noise, allowing observation of maternal behavior under chronic noise stress.

### Behavioral observations

Throughout the exposure period, both pups and dams were closely monitored for behavioral changes and signs of distress. Continuous observation during noise exposure sessions was achieved using a video surveillance system, allowing monitoring without disturbing the animals. Particular attention was paid to maternal behaviors, including nursing, grooming, and agitation, as well as to abnormal behaviors in pups once they became mobile (approximately at the end of the second postnatal week).

Qualitative observations included startle responses to the onset of music, maternal attempts to shield or relocate pups, escape-related behaviors (e.g., climbing or jumping attempts), and interactions among cage mates such as aggression or self-injurious behavior. General health indicators, including pup body weight gain and overt clinical signs (e.g., lethargy or seizures), were also recorded. Mortality was documented for any pups that died during the study, including the timing and circumstances of death.

An endpoint of maternal “behavioral despair or adaptation,” conceptually analogous to learned helplessness, was specifically evaluated. This was defined as the stage at which a dam, after initially exhibiting active escape or protective responses to noise exposure, ceased reacting and remained behaviorally passive during subsequent sessions. The number of dams exhibiting this demonstrated behavioral adaptation was recorded for each noise exposure group.

As pups’ eyes opened (around days 14–16), noise-exposed pups were more restless and exhibited delayed development of the righting reflex. The consistent behavioral responses to sound exposure (startle reactions and ultrasonic distress vocalizations) indicate preserved auditory responsiveness and argue against congenital hearing deficits.

### Tissue collection and histopathology

At the conclusion of the 30-day exposure period, all surviving pups were humanely euthanized for brain analysis. Deep anesthesia was achieved using sevoflurane inhalation, followed by intracardiac perfusion with cold heparinized saline and 10% neutral buffered formalin. The brains were quickly removed from the skull and immersion-fixed in 10% formalin for at least 7 days to ensure proper fixation. We then carefully dissected the temporal lobes to isolate regions of interest: this included the auditory cortex (temporal cortex), underlying hippocampus (with dentate gyrus), and the amygdaloid complex. These tissues were chosen based on our hypothesis and prior findings that noise can impact both auditory and limbic structures.

Fixed brain tissues were processed by standard paraffin embedding. Coronal sections (5 μm thickness) were obtained at the level of the dorsal hippocampus and amygdala (approximately corresponding to bregma −3.0 to −4.5 mm in the rat brain). Adjacent serial sections were prepared for different staining methods described below. Multiple sections from each brain region of each animal were analyzed to ensure sampling throughout the region’s extent.

For general histopathological evaluation, we stained sections with hematoxylin and eosin (H&E). This allowed us to assess overall morphology, the presence of any gross lesions (e.g., hemorrhages, edema), and the shape and condition of neuronal cell bodies. We paid particular attention to identifying degenerated neurons (characterized by dark, shrunken, or angular profiles with pyknotic nuclei) versus normal neurons (round, with vesicular nuclei and Nissl substance visible).

### Immunohistochemistry and TUNEL assay

To more specifically characterize neural injury, we performed immunohistochemical staining for several cellular markers and an apoptosis detection assay on additional sections:

- Neuron-specific enolase (NSE): NSE is a cytosolic enzyme present in mature neurons. Immunostaining for NSE helps to visualize neuronal cell bodies and can indicate neuronal integrity; loss of NSE immunoreactivity can be a marker of severe neuron injury or loss (Säljö et al., 2003). We used a mouse monoclonal anti-NSE antibody (clone E27, Roche/Ventana) at ready-to-use concentration.
- Glial fibrillary acidic protein (GFAP) and S-100β: GFAP is an intermediate filament protein in astrocytes, and S-100β is a calcium-binding protein found in astroglia and certain other glial cells. Both serve as markers of astrocytic activation and injury. In pilot tests, we found GFAP immunostaining effective for visualizing astrocyte morphology (e.g., fragmenting processes), and S-100 immunostaining was used in some sections to corroborate glial responses. Primary antibodies included a mouse monoclonal anti-GFAP and a mouse monoclonal anti-S-100 (clone 4C4.9, Roche Ventana), each used per manufacturer’s instructions (ready-to-use formulations).
- TUNEL (TdT-mediated dUTP nick end labeling) assay: This assay labels fragmented DNA in nuclei, a hallmark of late-stage apoptosis. We used the Roche *In Situ* Cell Death Detection Kit (POD) for TUNEL, which identifies apoptotic cells by enzymatically incorporating labeled nucleotides at DNA break sites. Sections were treated with proteinase K for antigen retrieval, then incubated with terminal deoxynucleotidyl transferase and labeled nucleotides. A peroxidase-conjugated antibody against the label allowed visualization via DAB (diaminobenzidine) chromogen, yielding a brown nuclear stain in apoptotic cells.

All immunohistochemical staining was performed on a fully automated IHC stainer (Leica Bond-Max system) to ensure consistency. The protocol included deparaffinization, rehydration, endogenous peroxidase quenching (3% H_2_O_2_ for 10 min), heat-mediated antigen retrieval (or enzymatic retrieval for certain epitopes as required), and incubation with primary antibodies (1 h at room temperature). Bound primary antibodies were detected with a biotin-free polymer detection system (Leica Bond Polymer Refine kit), which applies a secondary antibody-polymer HRP complex, followed by DAB chromogen development. Sections were counterstained lightly with hematoxylin. Appropriate positive controls (brain sections known to express the antigens) and negative controls (omission of primary antibody) were included to verify staining specificity.

Using these staining methods, we evaluated: (a) evidence of neuronal apoptosis (TUNEL-positive nuclei) and its distribution, (b) neuronal dropout or loss of normal neurons (by NSE staining and H&E morphology), and (c) astroglial activation or damage (GFAP/S-100 labeling patterns). Particularly, we noted that TUNEL reliably labeled dark brown nuclei in cells that also frequently showed shrunken eosinophilic cytoplasm on H&E, confirming they were degenerating neurons. GFAP staining in control brains showed delicate, long astrocytic processes, whereas in noise-exposed brains we looked for reactive changes such as thickened, fragmented astrocyte processes or astrocyte end-feet retraction around blood vessels.

### Unbiased stereological cell counting

To quantify neuronal degeneration, we employed stereological methods to estimate the numerical density of both normal and degenerated neurons in the defined regions (amygdala and dentate gyrus). We focused on these two regions as representative limbic structures heavily involved in stress and memory; both had clear boundaries in our sections and exhibited changes upon noise exposure.

**Figure 3:**
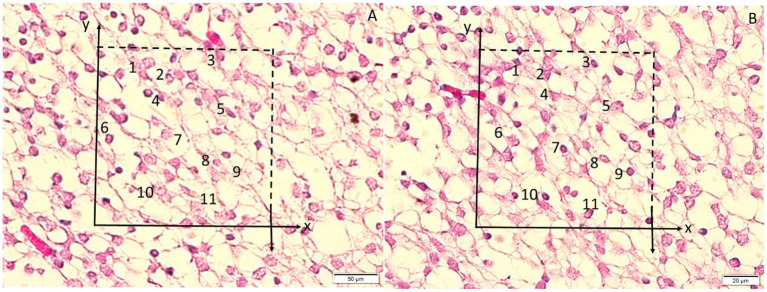
**(A,B)** Stereological counting of amygdala neurons using the physical dissector method. Micrographs A and B depict the same field of view captured from two adjacent sections separated by 5 μm. In the unbiased counting frame, the upper and right borders represent inclusion lines, whereas the lower and left borders (with extensions) represent exclusion lines. Neuronal nucleoli touching exclusion lines were excluded; nucleoli touching inclusion lines and located within the frame were counted as dissector particles unless their profiles extended into the reference section. Dissector particles are marked as 1, 3–5, and 10–11 in panel A; these profiles disappear in panel B. Nucleoli labeled 2 and 6–9 are not dissector particles in A. Numerical density was calculated using the formula: NvGN = ΣQ–GN/(t × A), where *t* is the 5-μm section separation and *A* is the counting-frame area.

We used an optical dissector/fractionator approach on NSE-stained sections to count neurons, distinguishing normal neurons from degenerated (apoptotic) neurons by morphological criteria and TUNEL co-labeling. Counting was done using a microscope equipped with a motorized stage and stereology software (Figures 3A,B). In brief, for each animal and each region, a series of systematically randomly sampled fields were analyzed. We defined neurons as “normal” if they had a large round cell body with an unstained (or lightly hematoxylin-stained) nucleus and visible nucleolus, and as “degenerated” if they were TUNEL-positive or showed condensed, dark nuclei with cell body shrinkage. The counting frame (a square of known area) was superimposed on each microscopic field, and neurons were counted if their nuclei were within the frame boundaries (using standard inclusion/exclusion lines to avoid edge effects). Using the optical dissector, we counted through the section thickness (taking two to three focal planes per section, with a disector height smaller than section thickness to avoid lost caps).

The numerical density of neurons (number of cells per cubic millimeter) was calculated using the following formula:


$$N=\Sigma Q^{-}/(\mathrm{\Sigma A}\times t)$$

In this equation, ΣQ^−^ represents the total number of neuron nuclei counted within all disector frames, ΣA is the total sampled area of the counting frames (in square millimeters), and t denotes the disector thickness (in millimeters). The Cavalieri principle was applied to estimate the volume of each brain region (such as the amygdala and dentate gyrus) through point counting on serial histological sections. The absolute total number of neurons or degenerated neurons in each region could then be obtained by multiplying the numerical density by the corresponding regional volume. However, in this study, neuronal densities were primarily reported, as they are independent of minor volumetric variations and more accurately reflect the relative proportion of affected neurons.

To minimize bias, an observer blinded to group identity performed the counting. The coefficient of error (Schmitz–Hof CE) was kept below 0.10 for the stereological estimates, indicating adequate sampling. Table 1 (in Results) summarizes the stereologically estimated apoptotic neuron densities for each group.

**Table 1:** Apoptotic neuron density (cells/mm^3^) in the amygdala and dentate gyrus across experimental groups (mean ± SD).

Brain region	Control	Single-speaker (85 dB)	Multi-speaker (85 dB)
Amygdala	1,321 ± 234	1,470 ± 285	3,460 ± 863 ★
Dentate gyrus	200 ± 34	7,600 ± 980 ★	13,450 ± 1,560 ★

★ *p* < 0.05 compared with control; the multi-speaker group also shows significantly higher apoptotic neuron density than the single-speaker group in both regions (*p* < 0.05; in the amygdala *p* < 0.005). (Values represent TUNEL-positive neuron nuclei per cubic millimeter, as estimated by stereological counting. Control values reflect natural developmental apoptosis. Single- and multi-speaker values reflect noise-induced apoptosis; multi-speaker exposure roughly doubled the apoptotic density vs. single-speaker in both regions).

### Statistical analysis

Data were analyzed using SPSS version 15.0 (SPSS Inc., Chicago, IL). For continuous variables (e.g., neuronal densities), we first tested for normal distribution and homogeneity of variances. Group comparisons of cell densities were made with one-way ANOVA followed by Tukey’s post-hoc tests if ANOVA was significant. In cases where only two groups were compared (e.g., single vs. multi-speaker for a particular outcome), Student’s *t*-test was used. Mortality rates and categorical outcomes (presence/absence of hemorrhage, behavioral adaptation, etc.) were compared using chi-square or Fisher’s exact tests as appropriate. Statistical significance was set at *p* < 0.05 (two-tailed). For key quantitative outcomes, we report group means ± standard deviation.

## Results

### Mortality and general health

During the 30-day exposure period, pup mortality was observed across all experimental groups. In the control group, three of six pups (50%) died, leaving three surviving pups available for histological analysis. In the single-speaker noise group, 5 of 15 pups (33%) died, whereas in the multi-speaker noise group, 8 of 20 pups (40%) died. Despite these numerical differences, a chi-square analysis revealed no statistically significant difference in mortality rates between groups (*p* = 0.78), noting the limited sample size in the control condition.

Most deaths in the noise-exposed groups occurred within the first two postnatal weeks, while in the control group, two of the three deaths occurred during the first postnatal week. All deceased pups were notably smaller than their littermates and exhibited poor weight gain prior to death. Routine necropsy or detailed histopathological examination of deceased pups was not performed due to the early postnatal timing of death and limited tissue availability; however, gross examination did not reveal any congenital abnormalities or overt signs of infection.

By postnatal day 30, surviving pups in both noise-exposed groups exhibited modestly reduced weight gain compared with controls. No gross congenital abnormalities, infections, seizures, or acute neurological deficits were observed in any group during the exposure period.

## Behavioral responses to noise

### Maternal behavior

Mother rats in both noise-exposed groups reacted strongly to music playback at the onset of the experiment. During the first several sessions, all dams displayed startle responses at music onset, rapid locomotion and attempts to escape the chamber, and behaviors interpreted as attempts to protect their pups (gathering pups to the nest or covering them). Mothers often assumed a vigilant posture with ears pinned back. Agitation episodes—circling the cage or climbing attempts—were frequent, and some dams exhibited excessive grooming or nipping at their pups. With continued exposure, a proportion of dams became less reactive: by the second week, approximately 60% of mothers in the single-speaker group and 50% in the multi-speaker group remained mostly still or passively huddled during noise sessions. The proportion of passive dams did not differ significantly between single- and multi-speaker groups (6/10 vs. 10/20; *p* = 0.26). Mothers in the multi-speaker group exhibited more frequent irritability and aggression outside exposure sessions; two dams occasionally bit their pups or gnawed cage bars. Weight loss of approximately 10–15% and poor fur condition were observed in 4 of 20 dams in the multi-speaker group and 2 of 15 dams in the single-speaker group. By the end of the experiment, some dams in the multi-speaker group had largely ceased typical maternal behaviors such as nursing and nesting.

### Pup behavior

During the first 12 days of life, behavioral differences among pups were minimal. As pups’ eyes opened (around days 14–16), noise-exposed pups were more restless and exhibited delayed development of the righting reflex and startle habituation relative to expected developmental milestones. During music playback in the latter half of the experiment, pups in the single-speaker group tended to crowd together or shelter beneath the dam, whereas pups in the multi-speaker group sometimes dispersed within the cage. There was no obvious difference in vocalizations; all noise-exposed pups occasionally emitted ultrasonic distress calls during loud sessions, while control pups did not.

### Distress and injury behaviors

Abnormal behaviors were observed in both noise-exposed groups. Two dams in the multi-speaker group inflicted minor bite wounds on themselves by the third week. In one case, a dam in the multi-speaker group cannibalized two weak pups during the first week. No such incidents occurred in the single-speaker or control groups. Disrupted sleep patterns were more common in the multi-speaker cages, with mothers and pups often awake or active at irregular times. Control group dams remained calm and exhibited normal rearing behavior.

## Histopathological findings

### Cerebral hemorrhages

**Figure 4:**
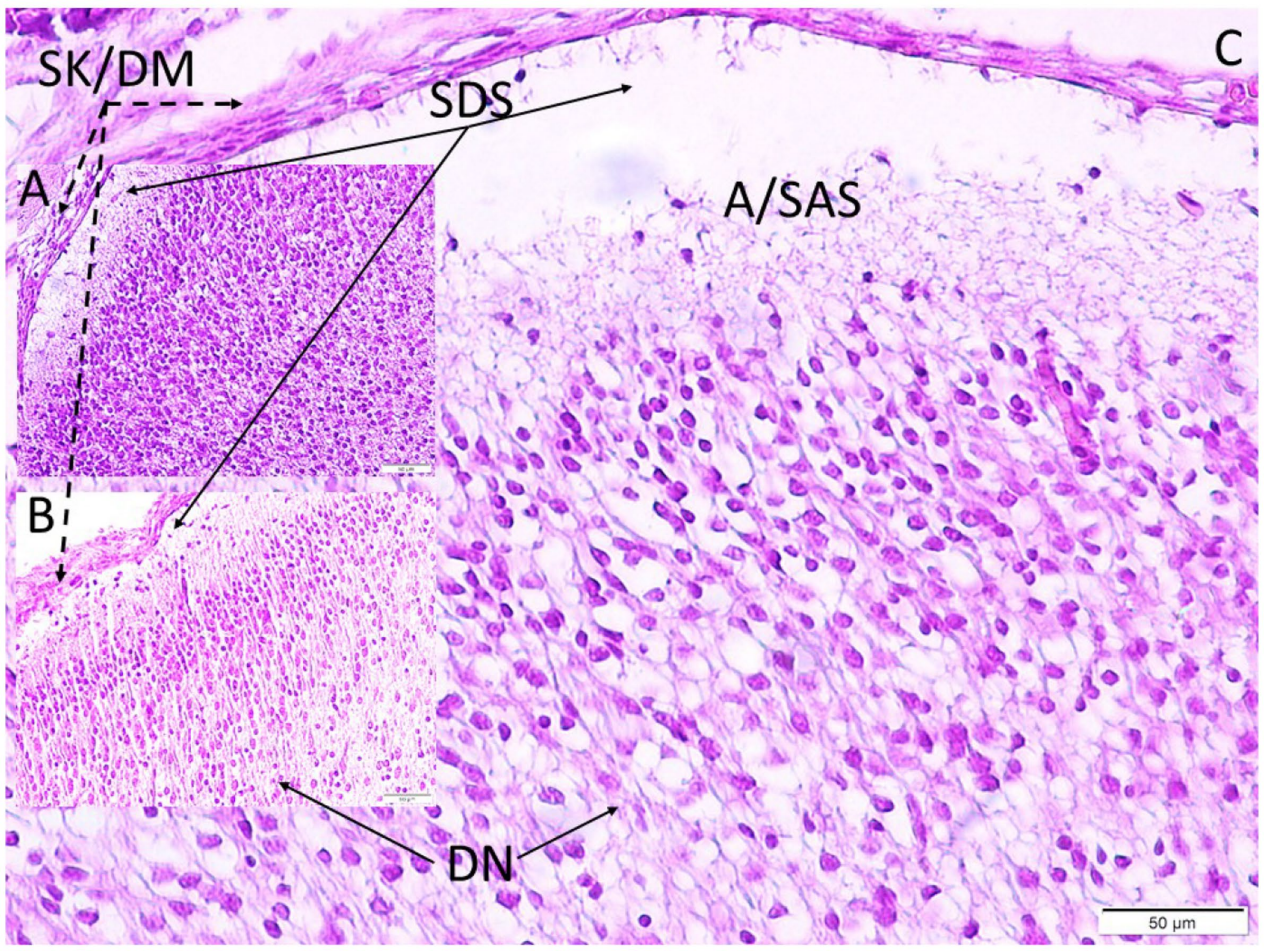
Figure **(A)** shows the skull and adjacent dura mater (SK/DM) of a newborn rat pup from the control group (GI), the subdural space (SDS) immediately below it, and the underdeveloped subarachnoid membrane and subarachnoid space (SAS) immediately below it. Figure **(B)** shows The subdural and subarachnoid spaces are observed to widen less in the group exposed to simple wave music (GII) and more in the group exposed to complex wave music (GIII) as shown in Figure **(C)**. The number of degenerated neurons in the temporal cortex of both groups also increases at the same rate (LM, H&E, x20).

After 1 month, brains from control rats appeared grossly normal. In the multi-speaker group, subarachnoid hemorrhages were observed in 5 of 12 surviving pups (42%), typically over the temporoparietal cortex; these appeared as patches of blood beneath the arachnoid and were confirmed microscopically by blood cell extravasation. In the single-speaker group, 1 of 10 pups (10%) showed a minor subarachnoid hemorrhage, and none occurred in controls. Cortical microhemorrhages were frequent in the multi-speaker group: 8 of 12 brains contained multiple small foci of erythrocyte leakage in cortical parenchyma, especially layers I–II of the auditory cortex, accompanied by dilated perivascular spaces and damaged capillary walls. Single-speaker brains contained far fewer petechial hemorrhages (3 of 10 brains, usually one or two small foci). Subarachnoid and subdural spaces were more expanded in the complex-wave–exposed brains than in controls (Figure 4).

### Neuronal morphology and degeneration

**Figure 5:**
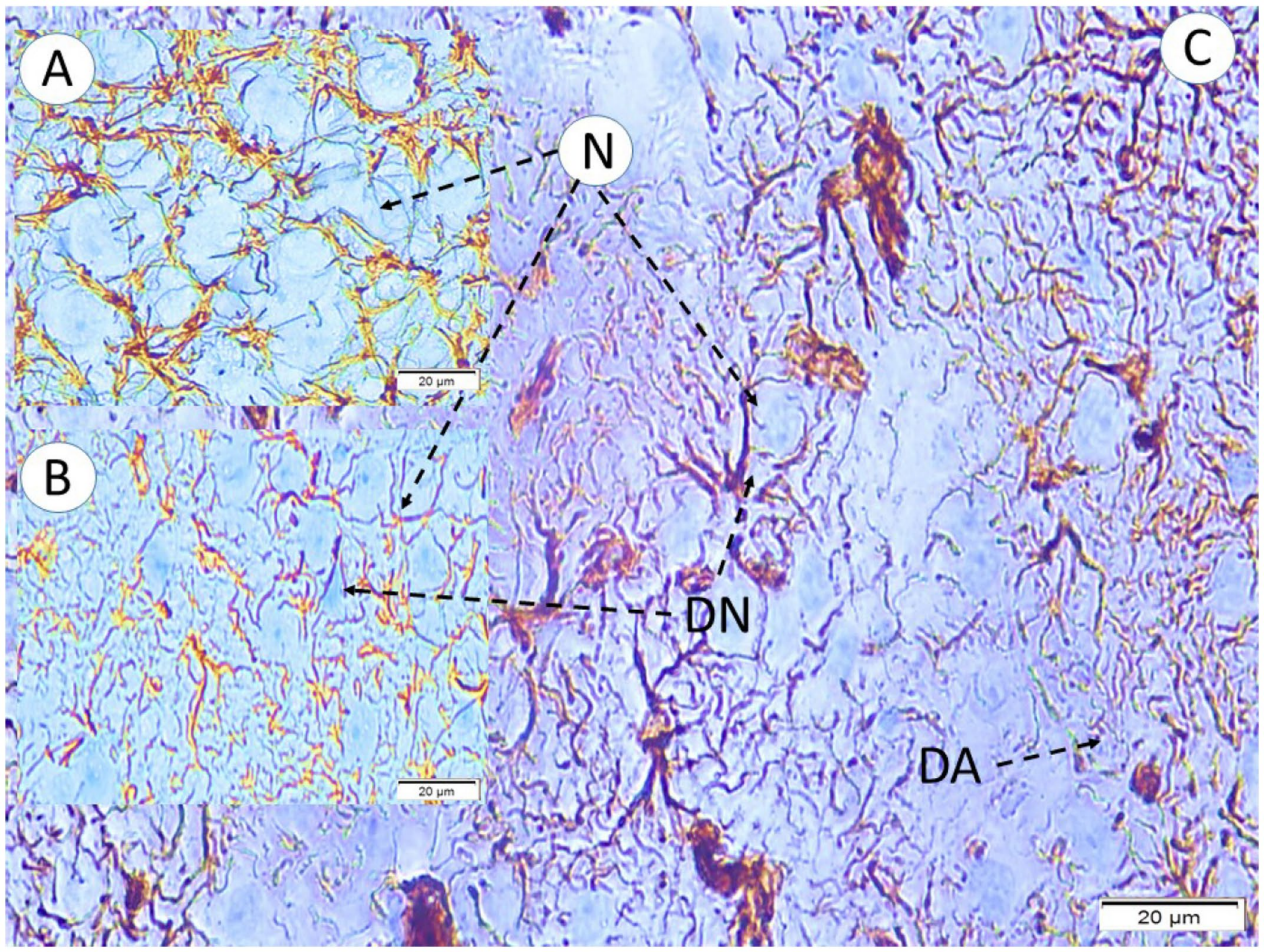
Neuronal and astrocytic morphology in the temporal cortex across experimental groups (LM, GFAP, ×40). **(A)** Control group (GI) showing normal neurons (N) and intact yellow-pink astrocytes. **(B)** Simple-wave exposure (GII) showing mild neuronal shrinkage and deformation, with astrocytes exhibiting slightly fragmented processes. **(C)** Complex-wave exposure (GIII) showing severe neuronal degeneration and deformation, accompanied by prominently fragmented, degenerated astrocytic processes (DA). Degenerated-neuron counts in the temporal cortex increase proportionally across the exposure groups.

In control rats, neurons in the temporal cortex, hippocampus and amygdala appeared normal, with round cell bodies, centrally located vesicular nuclei and prominent nucleoli; only occasional apoptotic figures were present. In noise-exposed rats, pathological changes were evident, particularly in the multi-speaker group. Neurons displayed shrunken, darkly eosinophilic cell bodies, irregular or elongated nuclei and perineuronal vacuolization (Figure 5). Many neurons showed angular deformity, with condensed cytoplasm and loss or peripheral aggregation of Nissl substance. In the single-speaker group, degenerated neurons were relatively sparse and scattered. In the multi-speaker group, these degenerated neurons were much more numerous: large clusters in the auditory cortex and temporal cortex (bordering the parietal lobe) were affected, and in some fields only a few normal-appearing neurons remained. The amygdala in multi-speaker rats showed widespread loss of normal neuronal appearance, with a relative paucity of intact neurons. NSE staining in multi-speaker rats demonstrated reduced neuronal immunoreactivity in affected areas.

### Apoptotic cell analysis (TUNEL assay)

TUNEL staining was used to quantify neuronal apoptosis across brain regions. In control animals, apoptotic activity was minimal, with mean densities of 1,321 ± 234 TUNEL-positive neurons per mm^3^ in the amygdala and 200 ± 34 per mm^3^ in the dentate gyrus.

In the temporal neocortex, apoptotic neuron density was significantly higher in the multi-speaker noise group (3,460 ± 863 per mm^3^) compared with the single-speaker group (1,470 ± 285 per mm^3^; *p* < 0.05). A similar pattern was observed in the amygdala, where apoptotic densities averaged 1,470 ± 285 per mm^3^ following single-speaker exposure and increased markedly to 3,460 ± 863 per mm^3^ following multi-speaker exposure (*p* < 0.005 versus both control and single-speaker groups).

**Figure 6:**
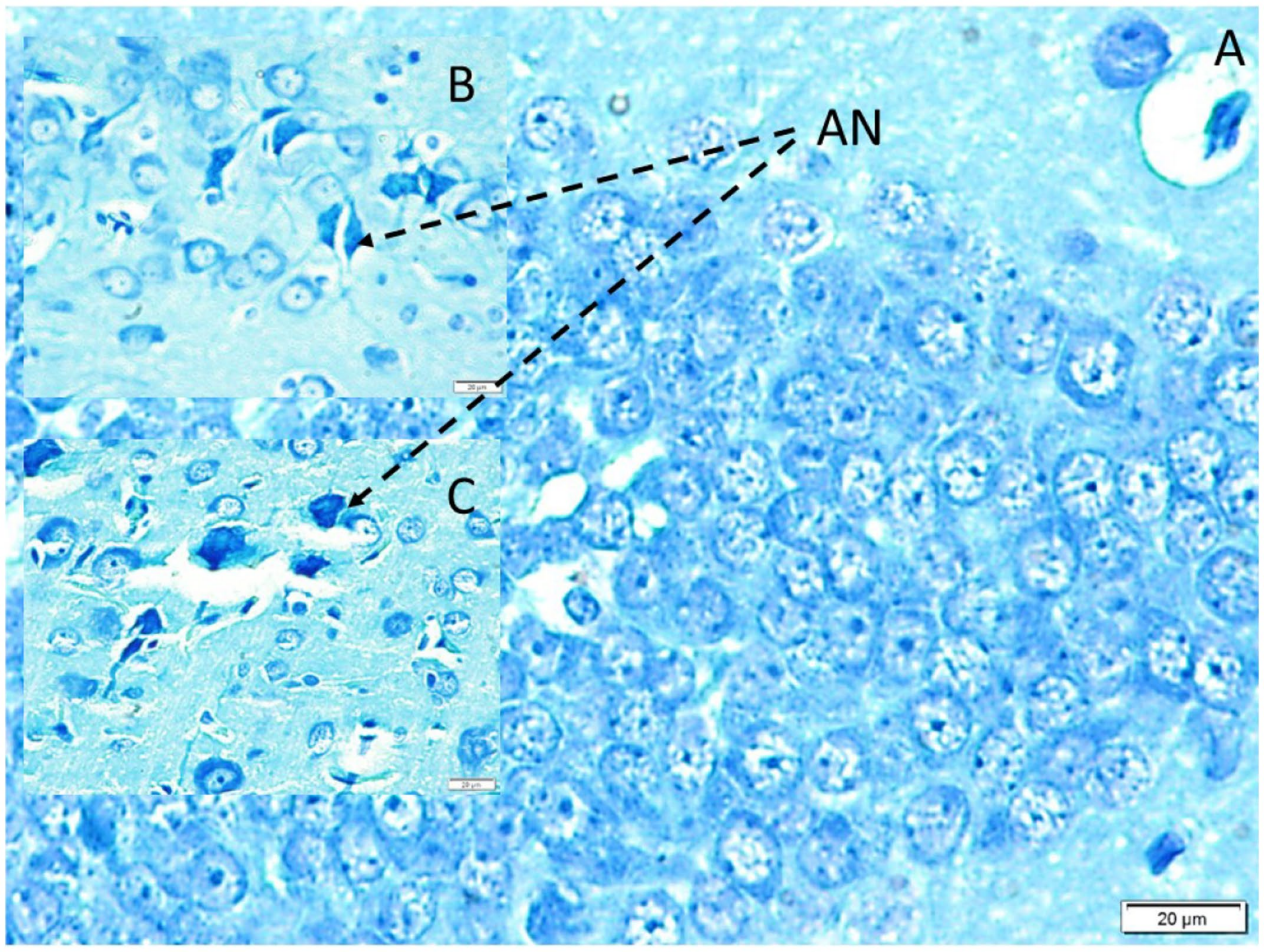
Apoptotic neuron distribution in the dentate gyrus (LM, GFAP, ×40). **(A)** Control group showing normal dentate gyrus architecture with very few TUNEL-positive nuclei, representing physiological developmental apoptosis. **(B)** Single-speaker (simple waveform) exposure showing a moderate increase in TUNEL-positive apoptotic neurons scattered within the dentate gyrus. **(C)** Multi-speaker (complex waveform) exposure showing a marked increase in TUNEL-positive apoptotic neurons, consistent with the quantitative stereological findings demonstrating significantly higher apoptotic neuron density compared with both control and single-speaker groups.

The most pronounced effects were observed in the dentate gyrus. Apoptotic neuron densities increased from 200 ± 34 per mm^3^ in controls to 7,600 ± 980 per mm^3^ after single-speaker exposure and further to 13,450 ± 1,560 per mm^3^ after multi-speaker exposure (*p* < 0.05 for multi-speaker versus single-speaker exposure; Table 1). Based on an estimated baseline neuronal density of approximately 123,000 neurons per mm^3^, these values correspond to degeneration of roughly 6–11% of dentate gyrus neurons in noise-exposed animals (Figure 6).

Throughout the manuscript, apoptotic neuron density refers to the stereologically estimated number of TUNEL-positive neuronal nuclei per mm^3^.

### Astroglial and blood–brain barrier changes

In control brains, GFAP immunostaining revealed thin, elongated astrocytic processes evenly distributed around neurons and blood vessels. Following single-speaker noise exposure, mild astrocytosis was observed, characterized by slightly thickened and shortened astrocytic processes. Occasional blood vessels exhibited subtle widening of perivascular spaces, while endothelial cell morphology remained largely preserved, consistent with only mild blood–brain barrier alterations.

**Figure 7:**
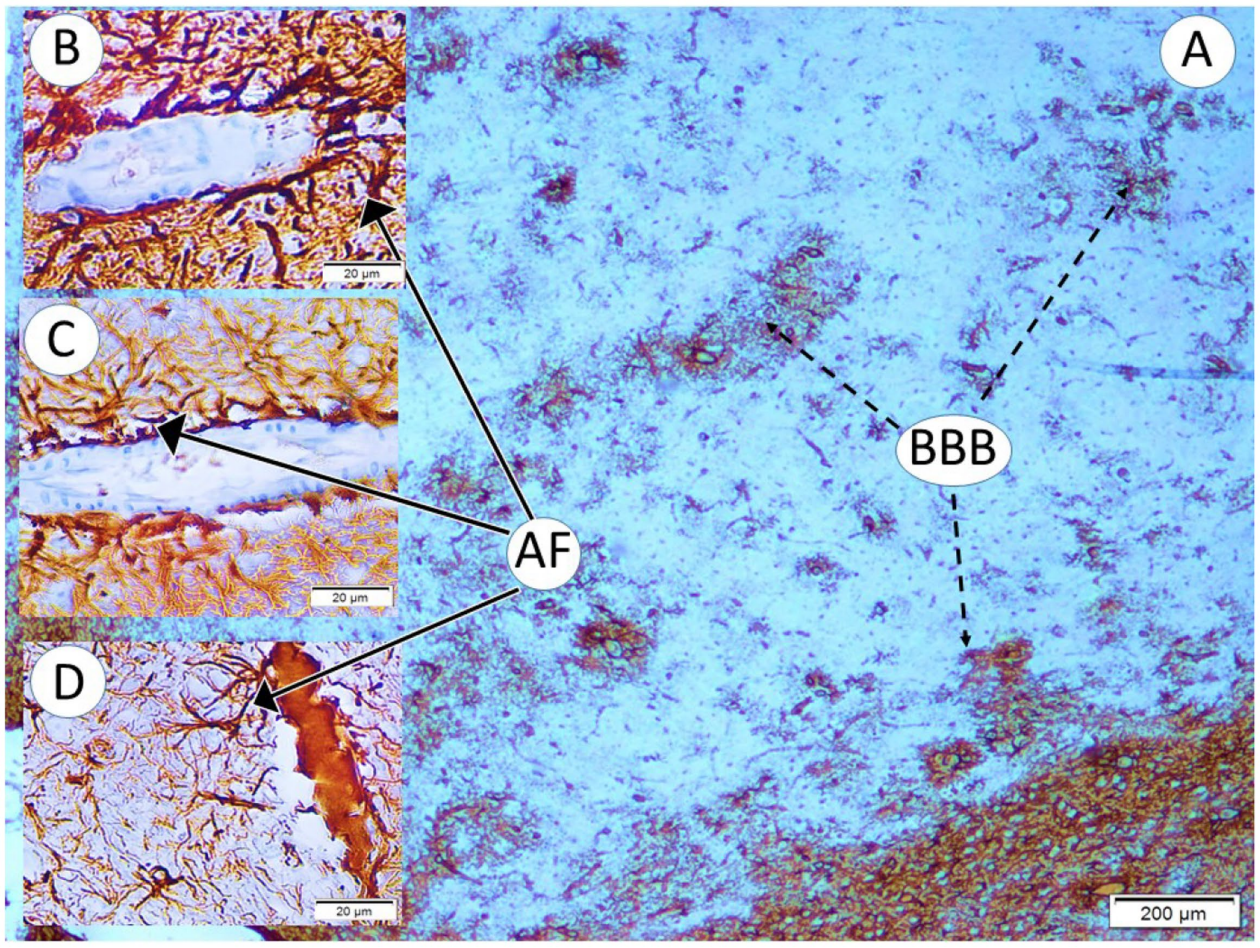
Blood–brain barrier and astrocytic changes in the temporal cortex under different acoustic conditions (LM, GFAP, ×4 for A; ×40 for B–D). **(A)** Normal BBB morphology in a control pup (GI). **(B)** Control arteriole with numerous intact astrocytic foot processes (AF). **(C)** Simple-wave exposure (GII) showing mildly degenerated endothelial cells and astrocytic processes with modest fragmentation. **(D)** Complex-wave exposure (GIII) showing severe endothelial degeneration, markedly fragmented and deformed astrocytic processes (AF) separated from the arteriole, and increased periarteriolar Virchow–Robin space.

In contrast, brains exposed to multi-speaker noise exhibited pronounced astroglial pathology. GFAP staining revealed severely disrupted astrocytic architecture, with truncated and beaded processes and occasional detachment of astrocytic end feet from capillaries. Vascular pathology was also prominent in this group: numerous microvessels displayed endothelial cell abnormalities, including swelling and structural distortion, accompanied by marked enlargement of perivascular Virchow–Robin spaces (Figure 7).

S100β immunostaining appeared more diffuse in the multi-speaker group, consistent with an enhanced astroglial response. This observation was qualitative in nature, as S100β levels were not quantitatively assessed in either brain tissue or serum in the present study.

## Discussion

This study demonstrates that complex multisource noise can inflict substantially greater neurodegeneration in the developing brain than a single-source noise of equal average intensity. Newborn rats exposed to an identical music track delivered through four loudspeakers, thereby generating an interference-rich acoustic field, exhibited more severe histopathological brain injury compared with those exposed to the same track from a single loudspeaker. Specifically, multispeaker exposure resulted in frequent subarachnoid and cortical microhemorrhages in the temporal lobes—lesions that were rare or mild in the single-speaker group and absent in controls—as well as a marked increase in neuronal apoptosis. Stereological analyses further demonstrated that the density of TUNEL-positive (apoptotic) neurons in key limbic regions approximately doubled under complex-waveform exposure relative to simple-waveform exposure. Although the number of surviving control animals was reduced due to natural neonatal mortality, mortality rates did not differ significantly between groups, and the observed histopathological differences were robust and region-specific, supporting the validity of the main findings.

Our results extend a growing body of evidence linking environmental noise exposure to neurotoxicity. Chronic or high-intensity noise has been associated with accelerated neurocognitive decline and structural brain alterations in both humans and animal models (Meng et al., 2022; Hahad et al., 2022). Epidemiological studies indicate that long-term residential or traffic-related noise exposure correlates with an increased risk of dementia and Alzheimer’s-type pathology in older adults (Weuve et al., 2021; Yu et al., 2020). In experimental models, intense acoustic trauma can induce diffuse brain injury characterized by blood–brain barrier disruption, neuroinflammation, increased neuronal membrane permeability, and apoptotic cell loss, resembling mild traumatic brain injury (Ma et al., 2024; Cui et al., 2018). Even moderate noise levels around 85 dB have been shown to trigger oxidative stress, tau and amyloid-*β* pathology, synaptic dysfunction, and impairments in learning and memory when exposure is chronic (Ma et al., 2024; Cui et al., 2015). Importantly, early developmental periods appear particularly vulnerable: prenatal or early postnatal noise exposure can impair hippocampal neurogenesis and spatial learning, while brief intense sound bursts may cause lasting structural reorganization within auditory pathways (Fernández-Quezada et al., 2025). Consistent with these findings, our data show that 1 month of daily exposure to 85 dB “disco” music delivered in a multisource configuration produces widespread neuronal degeneration in neonatal rats, underscoring the heightened susceptibility of the immature brain to acoustic stress. This increased vulnerability of the neonatal brain is inferred from developmental context and supported by existing literature, as the present study did not include adult animals for direct age-based comparison.

A key finding of the present study is that the severity of neural injury was substantially greater when the identical sound stimulus was delivered via multiple out-of-phase sources. From a physical acoustics perspective, simultaneous output from multiple loudspeakers generates complex interference patterns, producing spatially and temporally heterogeneous pressure peaks and troughs that differ fundamentally from the more uniform waveform generated by a single source. Experimental studies have shown that loud acoustic stimuli can transiently disrupt the blood–brain barrier, potentially through stress-mediated tight-junction disorganization and elevated circulating catecholamines (Semyachkina-Glushkovskaya et al., 2021; Sachdeva et al., 2022). In our model, although the average sound pressure level was maintained at approximately 85 dB SPL, multisource interference likely produced localized transient peaks exceeding this level, resulting in mechanical and vascular stress consistent with the subarachnoid hemorrhages and cortical microbleeds observed predominantly in the multispeaker group. Chronic noise exposure is also known to provoke neuroinflammation, astroglial activation, and neuronal loss in both auditory and extra-auditory brain regions (Cui et al., 2015; Wang et al., 2019). In addition, noise trauma can damage peripheral auditory structures such as the cochlea, impairing cochlear blood flow and inducing local inflammatory responses that may further amplify central neuronal vulnerability (Shin et al., 2019). As peripheral auditory structures were not examined in the present study, cochlear contributions to the observed central pathology cannot be excluded. Taken together, these mechanisms suggest that a multisource acoustic environment, combining peripheral auditory stress, vascular injury, blood–brain barrier disruption, and neuroinflammation, may impose a substantially greater neuropathological burden than an equivalent sound delivered from a single source.

The selective vulnerability of the amygdala and hippocampal dentate gyrus observed in this study is particularly noteworthy. These limbic regions play central roles in stress regulation, emotional processing, and memory formation and are well known to be sensitive to environmental stressors. Chronic or repeated noise exposure has been shown to impair hippocampal structure and function in rodent models, leading to deficits in learning and memory (Zhang et al., 2021). Previous studies have demonstrated that loud noise can damage hippocampal neurons, suppress neurogenesis, and induce synaptic and structural abnormalities within the hippocampus and related limbic circuits (Paciello et al., 2023; World Health Organization, Regional Office for Europe, 2018). In our study, both noise-exposed groups exhibited markedly increased neuronal apoptosis in the amygdala and dentate gyrus compared with unexposed controls, consistent with noise-induced excitotoxic or stress-hormone–mediated cell death. Notably, the multispeaker group showed approximately twice the apoptotic neuron density observed in the single-speaker group in both regions, indicating that acoustic interference exacerbates limbic neurodegeneration. These regions were selected *a priori* as regions of interest based on prior evidence, and although a comprehensive whole-brain pathology map was not generated, no comparable hemorrhagic or apoptotic lesions were observed outside the temporal cortex and limbic structures examined.

The nature of the acoustic stimulus warrants careful consideration. In the present study, noise exposure consisted of a commercially available “disco music” track containing both instrumental and vocal components, deliberately selected to approximate real-world recreational sound environments commonly encountered by humans. The use of mixed vocal–instrumental music was intended to enhance ecological validity by modeling typical music exposure scenarios rather than relying on an artificial instrumental-only stimulus. Our objective was not to disentangle neurobiological responses to specific sound categories (e.g., human voice versus instrumental sound), but to investigate the impact of acoustic waveform complexity generated by multisource interference under otherwise identical exposure conditions. Importantly, the same music track was used in all noise-exposed groups, and the sole experimental variable was the number of loudspeakers and the resulting acoustic interference pattern. Consequently, any potential stress-related effects attributable to vocal elements would have been equally present in both single-speaker and multispeaker conditions and therefore cannot account for the markedly greater neurodegeneration observed in the multispeaker group.

Maternal and pup behavioral observations further support the interpretation that chronic multisource noise imposes a substantial stress burden. Dams exposed to noise exhibited agitation, escape attempts, weight loss, disrupted sleep–wake patterns, and, in some cases, self-injurious behavior or impaired maternal care. Although maternal histopathology was not assessed, altered maternal behavior under chronic acoustic stress may have contributed to pup outcomes and should be considered a potential confounding factor. While behavioral outcomes in pups were not formally quantified, the pronounced limbic neurodegeneration observed raises concern that prolonged exposure to multisource noise during early development may have lasting consequences for emotional regulation, stress responsiveness, and cognitive function. Functional consequences of the observed anatomical injury, including long-term behavioral performance and auditory function, were beyond the scope of the present study.

Several limitations of this study should be acknowledged. First, the use of mixed instrumental and vocal music precludes conclusions about the relative contributions of specific sound categories, such as human voice, to stress or neurotoxicity. Second, the absence of adult comparison groups limits direct assessment of age-dependent vulnerability. Third, peripheral auditory structures, including the cochlea, were not examined, and their potential role in mediating central brain injury remains unresolved. Finally, long-term functional outcomes, including auditory thresholds and behavioral performance, were not assessed. Despite these limitations, the consistent and region-specific histopathological differences observed between single- and multispeaker exposure conditions provide strong evidence that acoustic waveform complexity is an important and previously underappreciated determinant of noise-induced neurodevelopmental injury.

From a public health perspective, these findings identify acoustic complexity as a critical factor in noise exposure risk that is not adequately addressed by current regulations. Existing guidelines primarily focus on average sound pressure level and exposure duration, without accounting for the number of sound sources or the resulting interference patterns (World Health Organization, Regional Office for Europe, 2018). In many real-world settings (such as concerts, nightclubs, and urban environments with multiple loudspeakers) individuals are routinely exposed to complex acoustic fields similar to the multispeaker condition studied here. Our data indicate that such environments may pose a greater neurodevelopmental risk than single-source noise at equivalent decibel levels. Given the heightened vulnerability of infants and young children, increased awareness and targeted mitigation strategies for multisource noise exposure in childcare settings, neonatal units, and public venues may be warranted.

## Conclusion

This experimental study provides novel evidence that complex multisource noise results in more extensive neurodegeneration in the neonatal brain than equivalent single-source noise exposure. Identical music, when played through multiple out-of-phase loudspeakers, produced greater neuronal death and vascular injury despite equal total sound intensity. These findings underscore that acoustic waveform complexity amplifies the neurotoxic effects of noise. Protecting developing brains may therefore require not only limiting noise intensity and duration but also recognizing the added risks posed by intersecting sound waves. Avoiding or buffering infants from environments with intense multispeaker noise is advisable. Further research is needed to explore the functional consequences of complex-noise–induced brain injury and to identify potential protective interventions, such as acoustic dampening strategies or pharmacological neuroprotection.

## Data Availability

The raw data supporting the conclusions of this article will be made available by the authors, without undue reservation.
